# Hematologic Chaos in Lupus Flare: A Case of Fulminant and Simultaneous Antiphospholipid, Anti-ADAMTS13, and Red Blood Cell Autoantibodies

**DOI:** 10.1155/2020/8812550

**Published:** 2020-11-13

**Authors:** Jafar Al-Mondhiry, Caroline Y. Chen, Michael H. Rosove

**Affiliations:** UCLA David Geffen School of Medicine, Los Angeles, CA, USA

## Abstract

Systemic lupus erythematosus may present with several distinct autoimmune phenomena simultaneously. We report a patient presenting with three serious hematologic disorders: thrombotic thrombocytopenic purpura, catastrophic antiphospholipid syndrome, and warm-type IgG red cell autoantibodies. The case is an example of the complex clinical nature of lupus and the importance of accurately identifying individual complications in order to optimize management.

## 1. Introduction

Systemic lupus erythematosus (SLE) is a mercurial and often morbid autoimmune condition characterized by indiscriminate antibody and immune complex formation. It may present with a broad range of hematologic complications. Without diagnostic clarity, critical therapeutic delays can result in significant morbidity. Herein, we describe a patient with SLE who presented in lupus flare with concurrent catastrophic antiphospholipid syndrome (CAPS), thrombotic thrombocytopenic purpura (TTP), and warm IgG red cell autoantibodies.

## 2. Case Description

A 38-year-old Hispanic woman with SLE presented to our emergency department with 2 weeks of headaches, visual disturbances, chest and abdominal pain, diffuse myalgias, and fever. Her lupus presented 8 years prior with progressive headache, altered mental status, and MRI brain showing multiple areas of enhancement with associated cortical edema, antinuclear antibody titer of 1 : 1280 (normal < 1 : 40) with homogenous pattern, anti-double-stranded DNA 243 IU/L (normal < 200), and an overall picture consistent with lupus cerebritis. On that same admission, she was also found to have thrombocytopenia, warm IgG autoimmune hemolytic anemia (WAIHA), positive rapid plasma reagin (RPR) at 1 : 8 with negative fluorescent treponemal antibody absorption (FTA-Abs), and “moderately positive” dilute Russell viper venom time (confirmatory tests performed but no ratio available), with no clinically evident thrombosis, prolongation of clotting times, or associated anti-beta2-glycoprotein antibodies (anticardiolipin antibodies were not checked). She was treated with prednisone, cyclophosphamide, and hydroxychloroquine with improvement in symptoms and cytopenias. She was lost to follow-up and was receiving no treatment at presentation.

On the current presentation, she was alert, with blood pressure 145/95 mm Hg, heart rate 125 beats per minute, temperature 38.1°C, oxygen saturation of 99% on room air, and weight 83 kg. Petechiae were present on bilateral distal lower extremities. No other rash, joint swelling/tenderness, alopecia, oral/nasal ulcers, lymphadenopathy, or hepatosplenomegaly were present.

**Figure 1:**
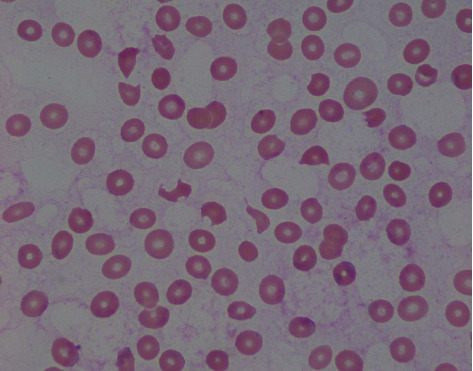
Blood smear (magnification 100x) showing numerous schistocytes and reduced platelets.

**Figure 2:**
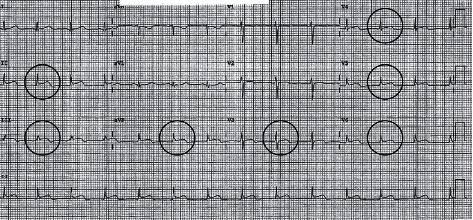
ECG having ST segment elevations in II, III, AVF, and V3 through V6.

**Figure 3:**
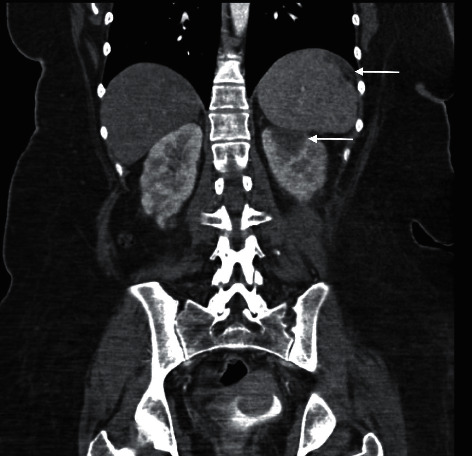
CT angiogram of the abdomen (arterial phase) showing left superior renal infarction and splenic infarction.

**Figure 4:**
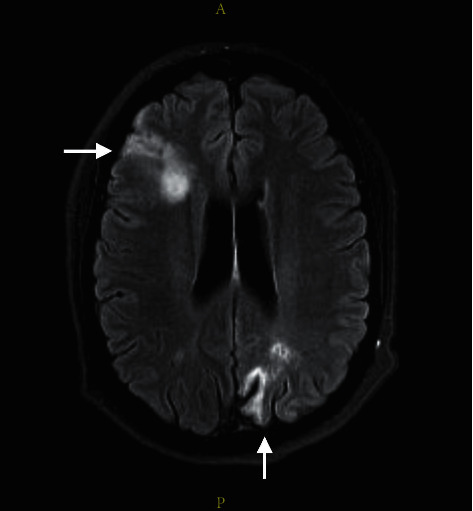
Contrast MRI brain (T2 FLAIR) showing right frontal subacute and left occipital chronic infarctions.

Immediately available findings included WBC 5.73 × 10^3^/*µ*L, hemoglobin 7.9 g/dL, mean corpuscular volume 82.4 fL, platelet count 14 × 10^3^/*µ*L, serum creatinine 0.88 mg/dL, total bilirubin 2.0 mg/dL, conjugated bilirubin 0.4 mg/dL, LDH 753 U/L (normal < 250), haptoglobin undetectable, reticulocytes 2.9%, PT/INR 1.1, aPTT 29.2 sec (normal 24.4–26.2), fibrinogen 692 mg/dL (normal 200–400), *C*-reactive protein 3.8 mg/dL (normal < 0.8), sedimentation rate 60 mm/hr (normal < 25), creatine phosphokinase 654 U/L (normal 38–282) serum ferritin 1151 (normal 20–180), and direct antiglobulin test 2^+^ positive for IgG and negative for C3. The blood smear showed frequent schistocytes and reduced platelets (Figure 1). A 12-lead ECG had ST elevations in leads II, III, AVF, and V3 through V6 (Figure 2), transthoracic echocardiography showed inferobasal hypokinesis, and serum troponin levels peaked at 37.8 (normal < 0.10), indicating acute myocardial infarction. Contrast CT imaging of the chest, abdomen, and pelvis and MRI brain showed infarctions of the left kidney, spleen, and brain (Figures 3 and 4).

**Figure 5:**
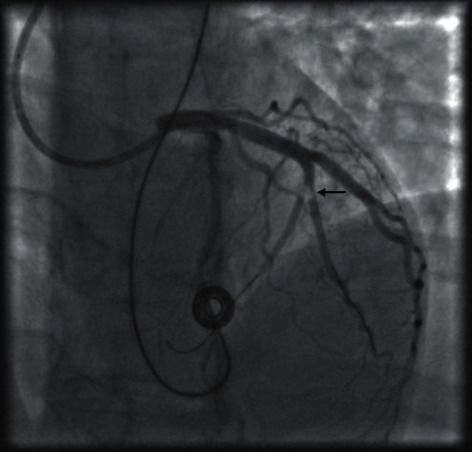
Cardiac catheterization showing occlusion of the left circumflex obtuse marginal branch (OM2).

Tentative simultaneous diagnoses of TTP and CAPS were made, and the presence of warm IgG red cell autoantibodies was additionally noted. The patient was given methylprednisolone 1,000 mg IV and transfused 4 units fresh frozen plasma (FFP) and 2 units of platelets en route to urgent cardiac catheterization and intervention, necessarily delaying full plasma exchange. In the cath lab, unfractionated heparin and aspirin were given. Thrombotic occlusion of the second branch of the left circumflex coronary artery was found, with no underlying coronary disease (Figure 5). Thrombectomy and balloon angioplasty restored normal blood flow. Full-dose unfractionated heparin by IV infusion was transitioned to full-dose twice-daily enoxaparin, and aspirin was continued. Methylprednisolone 1,000 mg IV was given daily for 2 more days and then prednisone 100 mg daily, in addition to hydroxychloroquine 200 mg twice daily. Plasma exchange was performed daily for 7 days. At the completion of exchange, one dose of rituximab 375 mg/m^2^ was given.

Over the first 48 hours, the following lab results returned: C3 68 mg/dL (normal 76–175), C4 10 mg/dL (normal 14–46), blood cultures negative, dilute Russell viper venom time (drawn before heparin administration) unequivocally positive with a confirmatory ratio of 1.9 (negative < 1.16) indicating a lupus anticoagulant, anticardiolipin IgM 35.3 (normal < 12.5), IgG negative, anti-beta2-glycoprotein IgM/IgG negative, and ADAMTS13 activity < 5%. No mixing study was performed due to aPTT being with reference range. Serologies were notable for negative anti-DS DNA, anti-SS-A/SS-B, anti-RNP, c-ANCA/p-ANCA, and anti-smooth muscle antibodies. Urinalysis and urine protein/creatinine were negative for proteinuria. No testing for severe acute respiratory syndrome coronavirus 2 (SARS-CoV-2) was performed, as her presentation predated the first identified case in the United States.

At hospital discharge seven days later, the platelet count was 272,000, hemoglobin 8.9, LDH 188, haptoglobin 93 mg/dL, and ADAMTS13 activity 62%. Two months later, the patient was clinically well and medications included prednisone 10 mg daily, hydroxychloroquine 200 mg twice daily, warfarin 15 mg daily (INR target range 2.5–3.5), and aspirin 81 mg daily. ADAMTS13 activity was 48% with only scattered schistocytes on blood smear. All presenting symptoms had resolved except for mild left visual field deficits. Unfortunately, the patient was again lost to follow-up, and antiphospholipid antibody testing could not be repeated after 12 weeks.

## 3. Discussion

Acquired TTP results from antibody-mediated inhibition of the von Willebrand factor (vWF) protease ADAMTS13. When ADAMTS13 activity is less than 5–10% of normal, the largest vWF multimers are not properly degraded, predisposing to formation of intravascular platelet thrombi and occlusion of terminal arterioles and capillaries. Brain and cardiac ischemias are common and typically produce the most life-threatening complications. Thrombocytopenia, hemolytic anemia with schistocytes, and ADAMTS13 activity less than 5–10% are uniformly present [1]. While most cases are spontaneous, about 7% of TTP cases arise in patients with SLE [2].

Emergency management of TTP includes plasma exchange, glucocorticoids, and, in severe or refractory cases, caplacizumab, a humanized anti-vWF monoclonal antibody preventing the association of vWF with platelets at the A1 domain [3]. Rituximab is often deployed after initial emergency management. Studies of recombinant ADAMTS13 are ongoing and may soon change this paradigm [4]. In this case, initiation of plasma exchange was necessarily delayed in order to prioritize coronary catheterization, and FFP served as a temporizing measure to supplement ADAMTS13.

Platelet transfusion in TTP is generally reserved for life-threatening bleeding or urgent invasive procedures due to the perceived risk of provoking further thrombotic events [5, 6]. Platelet transfusion in this case was necessary and apparently given without thrombotic complication.

Antiphospholipids (lupus anticoagulant, cardiolipin, and/or beta2-glycoprotein I antibodies) are commonly found in SLE and signify an elevated risk of thrombosis that may be large or small vessel, arterial, and/or venous. Our patient had a lupus anticoagulant, generally considered to have the strongest association with thrombosis when antiphospholipids are present, as well as cardiolipin antibodies of questionable specificity, given other potential inflammatory triggers for this low elevation of IgM antibodies. The size of the multiorgan infarctions in our patient (brain, heart, spleen, and kidney) suggest the vascular obstructions were significantly larger than the terminal arterioles affected by TTP, and such was decisively demonstrated at cardiac catheterization. The presence of a lupus anticoagulant 8 years prior further argues in favor of true antiphospholipid syndrome, and our patient appeared to satisfy all 4 criteria essential for the diagnosis of CAPS [7]. The management of CAPS is full anticoagulation with heparin and/or vitamin K antagonists, with anecdotal support for plasma exchange, corticosteroids, cyclophosphamide, and other immunosuppressants [8].

Our patient also had warm-type IgG red cell autoantibodies. Such antibodies are not a specific feature of either TTP or the antiphospholipid syndrome and thus are an independent autoimmune phenomenon. However, as hemolysis in our patient clearly occurred as a component of TTP and that spherocytes, the hallmark blood smear finding in WAIHA, were not present leave unclear how consequential the IgG red cell autoantibodies were.

Lupus patients offer a unique diagnostic challenge because they can present with more than one hematological derangement simultaneously, each of which may obscure the other. A high suspicion must be maintained for these morbid conditions in which appropriate management can significantly change clinical outcomes. This case reaffirms the age-old wisdom of Hickam's dictum – “A man can have as many diseases as he damn well pleases!” [9] – a lesson especially relevant to the care of patients with SLE.

## Data Availability

The patient data presented in this report are restricted by HIPAA in order to protect patient privacy and published here with the patient's consent. Additional data are available only by specific request from the corresponding author, Jafar Al-Mondhiry, MD, jalmondhiry@mednet.ucla.edu. The background data supporting the points made in the discussion are from previously published reviews, which have been cited in the text.

## References

[1] Joly B. S., Coppo P., Veyradier A. (2017). Thrombotic thrombocytopenic purpura. *Blood*.

[2] Deford C. C., Reese J. A., Schwartz L. H. (2013). Multiple major morbidities and increased mortality during long-term follow-up after recovery from thrombotic thrombocytopenic purpura. *Blood*.

[3] Saha M., McDaniel J. K., Zheng X. L. (2017). Thrombotic thrombocytopenic purpura: pathogenesis, diagnosis and potential novel therapeutics. *Journal of Thrombosis and Haemostasis*.

[4] Scully M., Knobl P., Kentouche K. (2017). Recombinant ADAMTS-13: first-in-human pharmacokinetics and safety in congenital thrombotic thrombocytopenic purpura. *Blood*.

[5] Swisher K. K., Terrell D. R., Vesely S. K., Kremer Hovinga J. A., Lämmle B., George J. N. (2009). Clinical outcomes after platelet transfusions in patients with thrombotic thrombocytopenic purpura. *Transfusion*.

[6] Goel R., Ness P. M., Takemoto C. M., Krishnamurti L., King K. E., Tobian A. A. R. (2015). Platelet transfusions in platelet consumptive disorders are associated with arterial thrombosis and in-hospital mortality. *Blood*.

[7] Asherson R. A., Espinosa G., Cervera R., Font J., Carles Reverter J. (2002). Catastrophic antiphospholipid syndrome. *JCR: Journal of Clinical Rheumatology*.

[8] Kazzaz N. M., McCune W. J., Knight J. S. (2016). Treatment of catastrophic antiphospholipid syndrome. *Current Opinion in Rheumatology*.

[9] Miller W. T. (1998). Letter from the editor: occam versus hickam. *Seminars in Roentgenology*.

